# Gene-Activated Matrix Comprised of Atelocollagen and Plasmid DNA Encoding BMP4 or Runx2 Promotes Rat Cranial Bone Augmentation

**DOI:** 10.1089/biores.2014.0057

**Published:** 2015-02-01

**Authors:** Mayumi Umebayashi, Yoshinori Sumita, Yousuke Kawai, Sumiko Watanabe, Izumi Asahina

**Affiliations:** ^1^ Department of Regenerative Oral Surgery, Unit of Translational Medicine, Graduate School of Biomedical Sciences, Nagasaki University, Nagasaki, Japan.; ^2^ Division of Molecular and Developmental Biology, Institute of Medical Science, The University of Tokyo, Tokyo, Japan.

**Keywords:** atelocollagen, *bmp4*, bone regeneration, gene-activated matrix, *in vivo*, *runx2*

## Abstract

To date, therapeutic method for *in vivo* gene delivery has not been established on bone engineering though its potential usefulness has been suggested. For clinical applications, an effective condition should be developed to transfer the genes *in vivo* without any transfection reagents or virus vectors. In this study, to facilitate the clinical setting of this strategy, particularly aimed at atrophic bone repair, we simply investigated whether manufactured gene-activated matrix (GAM) with atelocollagen containing a certain amount of plasmid (p) DNA encoding osteogenic proteins could augment the cranial bone in rat. GAMs were manufactured by mixing 0.02, 0.1, or 1 mg of AcGFP plasmid vectors harboring cDNA of BMP4 (pBMP4) or Runx2 (pRunx2) with 2% bovine atelocollagen and β-tricalcium phosphate granules. Before manufacturing GAMs, to determine the biological activity of generated pDNAs, we confirmed GFP expression and increased level of alkaline phosphatase activities in MC3T3-E1 cells transfected with pBMP4 or pRunx2 during culture. Then, GAMs were lyophilized and transplanted to onlay placement on the cranium. At 2 weeks of transplantation, GFP-expressing cells could be detectable in only GAMs containing 1 mg of AcGFP plasmid vectors. Then, at 4 weeks, significant bone formation was recognized in GAMs containing 1 mg of pDNAs encoding BMP4 or Runx2 but not in 0.02 or 0.1 mg of GAMs. These newly formed bone tissues surrounded by osteocalcin-stained area were augmented markedly until 8 weeks after transplantation. In contrast, minimal bone formation was observed in GAMs without harboring cDNA of osteogenic proteins. Meanwhile, when GAMs were transplanted to the cranial bone defect, bone formation was detectable in specimens containing 1 mg of pBMP4 or pRunx2 at 8 weeks as well. Thus, atelocollagen-based GAM reliably could form the engineered bone even for the vertical augmentation when containing a certain amount of plasmid vectors encoding osteogenic proteins. This study supports facilitating the clinical application of GAM for bone engineering.

## Introduction

At present, autogenous bone grafts, considered to be the gold standard, are especially used to regenerate bony defects in the craniofacial region.^1–3^ However, autografts involve donor-site morbidity and may lack osteogenic potential.^1,3^ Therefore, allografts or alloplastic bone substitutes, such as tricalcium phosphate (TCP), are often employed in clinics, but they lack osteoinductive properties.^1,3^ And so growth factors and morphogens have received increasing attention as materials that can confer osteoinducibility to allografts or alloplastic substitutes. In such growth factors and morphogens, bone morphogenetic proteins (BMPs), such as recombinant (r) BMP2 or 4, have been shown to induce bone formation in a variety of indications.^4–6^ However, direct implantation of high doses of rBMP2 is known to induce substantial swelling clinically that may cause the obstruction of airway when applied to oral and cervical areas.^7^ An efficient delivery method for the clinical use of osteogenic proteins remains to be developed.

As an alternative method for protein delivery, gene-activated matrix (GAM), which enables gene delivery safer and lower cost than protein, is showing the potential usefulness in tissue engineering.^8^ GAM comprises gene vectors encoding target proteins and proper biodegradable matrix such as collagen, and it can release gene vectors slowly to surrounding tissues and express proteins for long-term at physiological concentration after transplantation.^9–11^ In this system, the vectors for gene delivery can be generally divided into two classes: viral or nonviral vectors. Viral vectors can transfer genes very efficiently but there are several considerable disadvantages, such as immunogenicity, risks of virus-dependent recombination, or surplus protein expression exceeding the time period required for tissue regeneration.^8,12,13^ Therefore, nonviral plasmid vectors have been frequently employed to this system in various tissues though there were unsolved problems such as the low efficiency of transfection to induce the required tissue regeneration.^14–16^

To resolve these problems, development of GAM using nonviral vector has been attempted by adapting stem cells or using transfection reagents/kits.^17,18^ For instance, it has been demonstrated that mesenchymal stem cells (MSCs) combined with GAMs harboring osteogenic genes can increase the new bone formation *in vivo*.^19^ Moreover, GAM with transfection reagents/kits such as calcium phosphate (CaP) precipitate or polyethyleneimine (PEI) has been shown to be able to enhance the efficiency of plasmid gene transfer *in vivo*.^18,20,21^ However, though such improved methodologies can increase the efficiency of *in vivo* transfection, there may be a difficulty of obtaining a sufficient number of cultured MSCs or using the cytotoxic transfection reagents for GAM system clinically. Thus, efficient methods for generating GAM for bone engineering have not yet been established. Nonviral GAM with a certain transgene efficiency and low toxicity should be developed.

Meanwhile, improving matrix material is also an essential matter for developing the nonviral GAM. Previously, original GAM combined plasmid (p) DNA to collagen matrix, which was developed by Bonadio et al., and showed the low efficacy of gene transfer in rat boney defect.^10^ Therefore, various biomaterials, such as alignate, chitosan, or gelatin, have been investigated as carrier matrixes for nonviral GAM to date.^22^ As an example of recent progress in such candidates of matrixes, an atelocollagen-based nonviral delivery method has been demonstrated to be a reliable approach to achieve the maximal function of nucleotides, such as pDNA and antisense oligonucleotide, *in vivo* via local administration.^23^ Atelocollagen, a highly purified collagen without the terminal peptides, is a biomaterial with low antigenicity and high bioaffinity, and has been widely used clinically as an implantable material. This material can release pDNA slowly for long-term with appropriate dose in natural body.^23–25^ Therefore, atelocollagen is considered as a most potent candidate of matrix for nonviral GAM. In fact, previous studies have shown the potential of atelocollagen-mediated antisense therapeutics for the treatment of cancer or inflammatory disease.^25,26^ However, though this material can be employed for clinical treatments immediately, pure effectiveness of atelocollagen has not been well examined as a matrix of nonviral GAM for bone engineering without stem cells or transfection reagents/kits.

The aim of this study is to have a new understanding on the usefulness of delivering nonviral GAM without cell transplantation or any transfection reagents/kits for facilitating clinical setting of bone engineering. To take a step toward establishing simple and safe concept for GAM, we first confirmed whether atelocollagen-based GAM containing a certain amount of naked pDNA encoding osteogenic proteins was superior in bone engineering. Currently, administration of pDNA is considered to be safe *in vivo*, even if employed at high dose to a certain extent.^27,28^ Therefore, to facilitate the clinical setting of GAM for bone engineering, *in vivo* delivery of naked pDNA using only atelocollgen should be taken a fresh look as a simple and low-toxic method even if its transfection efficacy is not so conspicuous compared with that of attempting cell transplantation or transfection reagents/kits. Meanwhile, it is known that lyophilized atelocollagen can also act as a 3D scaffold for bone engineering.^29^ Therefore, we hypothesized that lyophilized GAM, which consists of atelocollagen and pDNA encoding effective osteogenic proteins such as BMP4 or Runx2, could reliably induce the bone formation. To clarify the regenerative capability of this simple and probably safe GAM, we transplanted it to onlay placement on rat cranium. We employed this bone augmentation model in this study as a definitive model of bone engineering because the severe alveolar bone atrophy is one of the major obstacles in oral and maxillofacial area and the novel osteo-inductive bioartificial materials are required strongly to be developed without autologous bone graft.

## Materials and Methods

### Plasmid preparation

All procedures with animals were carried out under the protocol approved by the Facility Animal Care Committee of Nagasaki University. All expression vectors were constructed using the standard recombinant PCR method and confirmed nucleotide sequencing. Both mouse (m) *bmp4* and *runx2* cDNAs were obtained from embryo 18.5 mouse calvaria. Total RNA was extracted with TRIzol (Invitrogen, Carlsbad, CA), and reverse transcription was performed according to the manufacturer's instruction (ReverTra Ace^®^qPCR RT Master Mix with gDNA Remover; Toyobo, Osaka, Japan) with specific primer sets constructed using NCBI reference sequence (BMP4; NM_007554.2, Runx2; NM_001145920.2). Forward primers involved Kozak sequence and *Xho*I site, and reverse primers included *Sal*I site for ligation (*bmp4* primer pair: forward 5′-ctcgaggccaccatgattcctggtaacc gaatgc-3′ and reverse 5′-gtcgactcagcggcatccacaccc-3′; *runx2* primer pair: forward 5′-ctcgaggccaccatgcgtatt cctgtagatcc-3′ and reverse 5′-gtcgactcaatatggccgccaaac agactcatccattctgc-3′). pAcGFP-N1 vector (Clontech, Palo Alto, CA) connected with IRES site (pIRES-AcGFP; Kindly supplied by Dr. T. Komori, Nagasaki University, Japan) was prepared. After pIRES-AcGFP vector and cDNAs were digested using *Xho*I and *Sal*I, vector and cDNAs were ligated. All expression plasmids newly generated in this work were verified by DNA sequencing (BigDye^®^ Terminator v3.1 cycle Sequencing Kit; Invitrogen).

### Transfection to MC 3T3-E1 and osteoblastic differentiation

MC3T3-E1 cells were cultured in α-MEM (Wako, Osaka, Japan) supplemented with 10% fetal bovine serum, 0.6 mg/mL glutamine, and 2% antibiotic–antimyotic (Gibco, Grand Island, NY) in 10 cm dishes at 37°C in 5% CO_2_. After confluence, cells were harvested and resuspended into 100 μL Resuspension Buffer R (Invitrogen) at a density of 5×10^5^ cells/mL, and mixed with 20 μg constructed plasmids of each gene. Then cells were transfected with pAcGFP (pGFP), pAcGFP-BMP4 (pBMP4), or pAcGFP-Runx2 (pRunx2) with Neon (Invitrogen) using No.16 program (1400 voltage, 20 ms, 2 pulses). After transfection, cells were seeded on a 6-well plate and cultured for 7 days. The next day after transfection, cells were analyzed for GFP transfer by a fluorescence microscope (ECLIPSE; Nikon, Tokyo, Japan), and the efficacy of transfection was analyzed with NIH ImageJ software (NIH, Bethesda, MD). The percentage of surface area occupied by GFP signals was assessed under ×200 magnification using five images from each of the three wells per group. Two examiners independently took photos randomly, and then the expression areas were measured by pixels. During the culture, cell proliferation and alkaline phosphatase (ALP) activities were measured at 4 and 7 days.

Cell proliferation was measured using WST-8 kit (Dojindo, Kumamoto, Japan) according to manufacturer's protocol. Briefly, cells were incubated with a medium containing 100 μL/mL of WST-8 for 1 h. The absorbance was read on a spectrophotometer at 450 nm (Multiscan FC; Thermo Scientific, Waltham, MA). ALP activities were measured according to the method of Lowry (1955). An aliquot of supernatant was added to p-nitrophenylphosphate containing MgCl_2_ (Sigma-Aldrich, St. Louis, MO) and the mixture was incubated at 37°C for 15 min. NaOH of 0.2 N was added to stop the enzymatic reaction and absorbance was read at 405 nm with a spectrophotometer. ALP activity is expressed as μmol p-nitorphenol/cell. Each experiment was performed in triplicate for three samples.

### Preparation of GAM

GAMs used in all experiments were prepared the day before transplantation. An amount of 0.02, 0.1, and 1 mg of AcGFP plasmid vectors harboring cDNA of BMP-4 or Runx2 were dissolved in 60 μL sterilized water and mixed well with 100 μL of 2% bovine atelocollagen (Atelocollagen Implant; Koken, Tokyo, Japan) and 20 mg β-TCP granules (Osferion; Olympus, Tokyo, Japan) at the inside of lids of 1.5 mL Eppendorf tubes. Then, these mixtures were freeze-dried overnight (Fig. 2A). In this study, the vehicle AcGFP plasmids only were employed as experimental controls. Experiments were performed in each experimental group such as, transfected with nonplasmid vectors (controls; Contl), transfected with only pGFP vectors (GFP), transfected with pBMP4 (BMP4), and transfected with pRunx2 (Runx2).

### GAM transplantation

Six- to seven-week-old male rats (F344; Clea, Tokyo, Japan) were anesthetized with an intraperitoneal injection of sodium pentobarbital (15 mg/kg; Nembutal; Dainippon Sumitomo Pharma, Osaka, Japan). During and after surgery, rats were kept warm. Then, GAMs were transplanted to the cranial bone surface under the periosteum of the F344 rats (*n*=171; 6 rats per group [each group of GFP, BMP4, and Runx2 containing 0.02, 0.1, or 1 mg pDNA, respectively] at each time point [2, 4, and 8 weeks after transplantation], plus 3 rats in Contl group at each time point) as a bone augmentation model (Fig. 2A). At 2, 4, and 8 weeks after transplantation, specimens were harvested to evaluate the efficiency of transfection and new bone formation by histological/immunohistological analysis. Moreover, to confirm the effectiveness of GAM further, critical size of circular calvarial defects (diameter, 9 mm) was created using a saline-cooled trephine drill as a bony defect model, and then individual GAMs were transplanted into the bony defects of male F344 rats (*n*=18). At 8 weeks of transplantation, specimens were harvested for histological analysis.

### Detection of *in vivo* gene transfer

To confirm the presence of transfected cells after 2 weeks of transplantation, frozen sections (5 μm thickness) of harvested transplants (only transplanted GAM) were fixed with 4% paraformaldehyde, and then GFP signals in specimens were observed by using a confocal laser scanning microscope (Zeiss, Jena, Germany) at 488 nm excitation.

### Histological and immunohistological analysis

To assess the new bone formation after 4 and 8 weeks of transplantation, harvested specimens were fixed with 4% paraformaldehyde; decalcified with a solution containing 2.9% citric acid, 1.8% tri-sodium citrate dehydrate, 10% formic acid, and 90% distilled water; and embedded in paraffin wax. Sections (3 μm thickness) were deparaffinized and stained with hematoxylin and eosin. The volume of newly formed bonelike tissues was analyzed with NIH ImageJ software (NIH). The percentage of surface area occupied by bonelike tissues was assessed by light microscopy under ×30 magnification using five sections from each of the six specimens per group. Two examiners independently chose sections randomly and then the new bone areas were measured by pixels. Then, immunohistochemical staining for the specimens at 8 weeks of transplantation was performed with Vectastain ABC kit (Vector, Burlingame, CA). Sections were stained with mouse monoclonal anti-rat osteocalcin (1:200; Abcam, Cambridge, UK), and the slides were incubated with an anti-mouse secondary antibody (1:200). Then, specimens were finally reacted with 0.1%w/v 3.3′-diaminobenzidine tetrahydrochloride (DAB immunohistochemistry; GenWay) in PBS and counterstained with hematoxylin. Control staining was performed by replacing the first antibody with preimmune serum eluted from the corresponding affinity columns.

### Statistical analysis

Means were analyzed using one-way analysis of variance. Dunnett's multiple comparison *t*-test was used to detect any significant differences within each group. Experimental values were presented as mean±s.d. A *p*-value of <0.05 was considered to be statistically significant.

## Results

### Biological activity of generated plasmid vectors

**FIG. 1.**
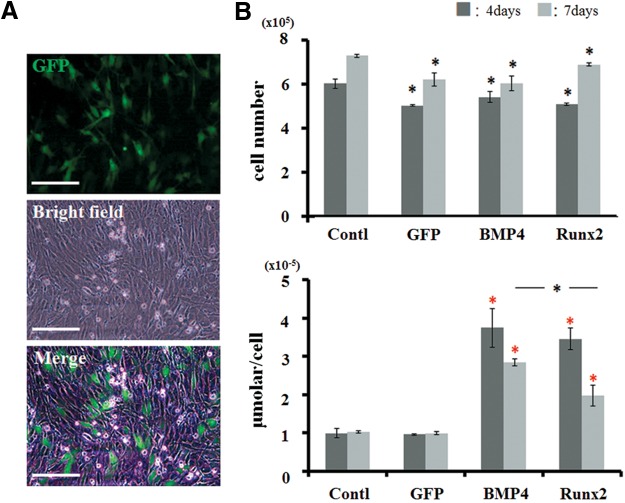
Biological activity of generated plasmid vector. **(A)** Expression of GFP signals in MC3T3-E1 cells after 24 h of transfection. Scale bar is 50 μm (200×). **(B)** Changes of cell proliferation and ALP activity at 4 and 7 days after transfection. Total cell number did not show a significant difference across all transfected cell groups. BMP4 and Runx2 groups showed the increased activities of ALP. Black asterisks in the graph of cell proliferation represent statistical significance between the control group and the transfected cell groups at 4 or 7 days (*p*<0.05). Red asterisks in the graph of ALP activity represent statistical significance between the GFP group and the experimental groups at 4 or 7 days (*p*<0.01). Black asterisks in the graph of ALP activity represent statistical significance between BMP4 and Runx2 groups at 7 days (*p*<0.05). ALP, alkaline phosphatase; BMP4, pBMP4-transfected cell group; Contl, control nontransfected cell group; GFP, pGFP-transfected cell group; Runx2, pRunx2-transfected cell group.

We first analyzed the biological activity of generated pDNAs for osteoblastic differentiation *in vitro*. After 24 h of transfection, a certain number of MC3T3-E1 cells expressed GFP signals (which occupied ∼30% of total area) (Fig. 1A). There were no differences in this efficacy among groups (GFP, BMP4, and Runx2). Then, when the cells were cultured until 4 or 7 days, total cell number did not show a significant difference across all transfected cell groups (Fig. 1B) though that number in the control group was significantly higher than that in the treated groups. However, the cells transfected with pDNAs encoding BMP4 or Runx2 (BMP4 or Runx2 groups) showed the increased activities of ALP on both day 4 and day 7, with most significant differences on day 4 (Fig. 1B). At day 7 day, ALP activities in both BMP4 and Runx2 were decreased, while the BMP4 group remained at a statistically higher level of ALP compared with the Runx2 group. In contrast, the cells transfected with nonencoding pDNA (GFP group) did not show any changes of this activity during the culture. We determined that generated pDNAs encoding BMP4 or Runx2 have biological activities for osteoblastic differentiation without certain cytotoxicity.

### Detection of transfected cells *in vivo*

**FIG. 2.**
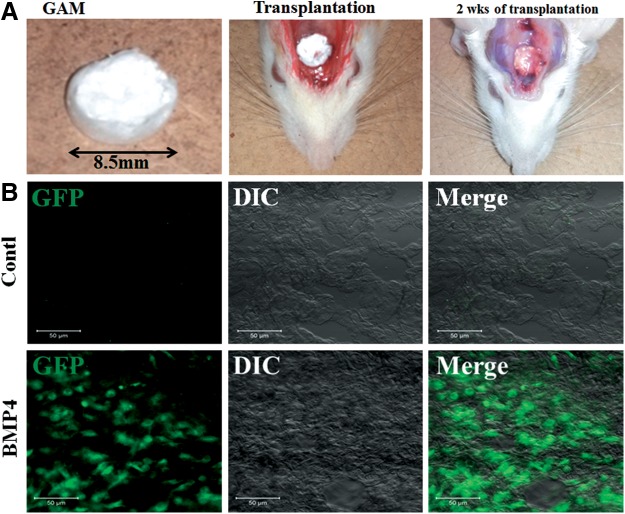
GAM transplantation and detection of *in vivo* gene transfer. **(A)** Gross appearance of manufactured GAM, just transplanted GAM to cranial bone surface, and transplanted GAM after 2 weeks. **(B)** GFP expression of migrated cells in the surface area of GAM after 2 weeks of transplantation. Representative images in GAM of BMP4 group are shown. Expression was recognized only in GAM harboring 1 mg of pDNA (GFP, BMP4, and Runx2) groups. DIC, differential interface contrast; GAM, gene-activated matrix. Scale bar is 50 μm.

AcGFP expression from the AcGFP-N1 plasmid on rat cranium was assessed at 2 weeks after the transplantation of manufactured GAM (Fig. 2A). GFP expression of transplants was recognized in the surface area of GAM harboring 1 mg of pDNAs (GFP, BMP4, or Runx2 groups) by a confocal laser scanning microscope (Fig. 2B). However, when GAM contained 0.02 and 0.1 mg pDNAs, we could not find obvious GFP expression in specimens (data not shown). Likewise, no expression could be detected in GAM without pDNA (control group).

### Histological analysis

**FIG. 3.**
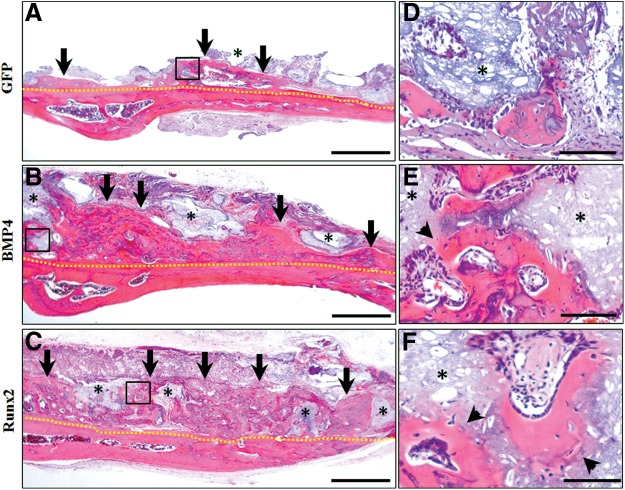
Histological observation at 4 weeks of GAM transplantation to onlay placement. **(A–C)** Representative images of hematoxylin and eosin (HE) staining of specimens in GFP, BMP4, and Runx2 groups, respectively. Considerable bone formation was recognized along the host bone in BMP4 **(B)** and Runx2 **(C)** groups when compared with that in GFP **(A)** group. Scale bar is 50 μm. **(D–F)** The black box areas in **(A–C)** are shown in higher magnification. Newly formed bone in BMP4 **(E)** and Runx2 **(F)** groups clearly surrounded β-TCP granules, while absorption areas of β-TCP granules could not be found obviously in the GFP group **(D)**. Scale bar is 10 μm. Yellow dotted line indicates boundary of the host and newly formed bone; black arrow, area of augmented bone; asterisk, β-TCP granules; and black arrow head, replacement to bone at the surface of absorbed β-TCP granules.

The histology of the rat cranium (onlay placement) at 4 weeks postoperatively is shown in Figure 3. When GAMs harboring 1 mg pDNAs were transplanted, new bone formation was found in all groups (GFP, BMP4, and Runx2 groups) (Fig. 3A–C). However, while small amounts of new bone were seen in the immediate proximity of host bone in the GFP group, considerable bone formation was recognized along the host bone in BMP4 and Runx2 groups. Newly formed bone in BMP4 and Runx2 groups clearly surrounded the β-TCP granules, and also bone marrows were formed abundantly in the augmented area (Fig. 3B,C). Furthermore, replacement to bone tissues, which included osteocytes, was observed at the surface of absorbed TCP granules on the magnified micrographs (Fig. 3E,F). In contrast, absorption areas of TCP granules could not be detected obviously in the GFP group (Fig. 3D). On the other hand, only small amounts of new bone formation similar to the GFP group containing 1 mg pDNAs (Fig 3A) were detectable when transplanted GAM contained 0.02 or 0.1 mg pDNAs (data not shown).

**FIG. 4.**
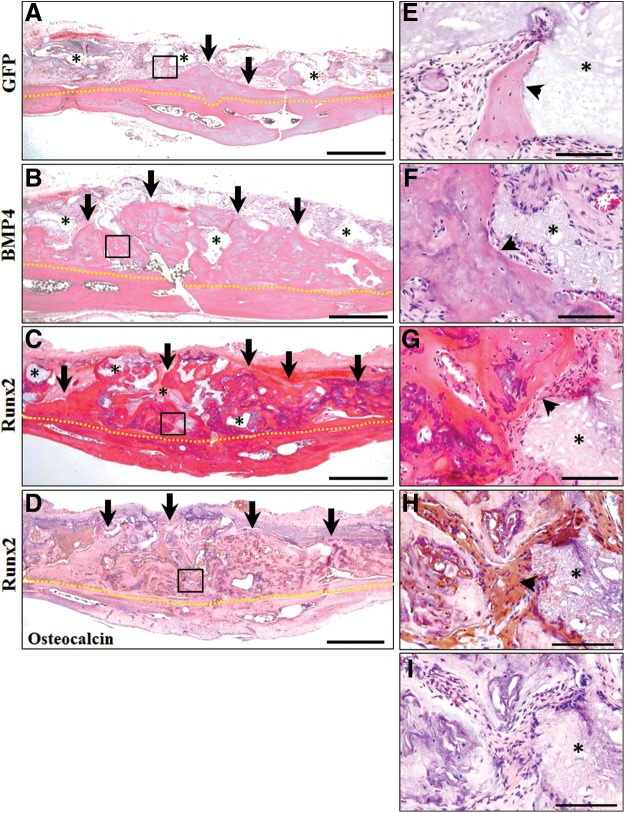
Histological observation at 8 weeks of GAM transplantation to onlay placement. **(A–C)** Representative images of HE staining of specimens in GFP, BMP4, and Runx2 groups, respectively. New bone area was markedly augmented in specimens of BMP4 **(B)** and Runx2 **(C)** groups while small amounts of new bone detectable in the GFP group **(A)**. Scale bar is 50 μm. **(D)** Representative images of osteocalcin immunostaining in the Runx2 group. Scale bar in 50 μm. **(E–I)** The black box areas in **(A–D)** are shown in higher magnification. Mature bone tissues, which included osteocytes, were observed in BMP4 **(F)** and Runx2 **(G)** groups, while small amounts of new bone formation were recognized in close proximity to β-TCP granules in the GFP group **(E)**. Osteocalcin-positive cells were seen at the surface of new bone tissues **(H)** and not recognized in control sections treated with preimmune serum (negative control) **(I)**. Scale bar is 10 μm. Yellow dotted line indicates boundary of the host and newly formed bone; black arrow, area of augmented bone; asterisk, β-TCP granules; and black arrow head, replacement to bone at the surface of absorbed β-TCP granules.

The histology of the rat cranium (onlay placement) at 8 weeks postoperatively is shown in Figure 4. At this stage, only small amounts of new bone were detectable in the GFP group containing 1 mg pDNAs (Fig. 4A) and GAMs containing 0.02 or 0.1 mg pDNAs (data not shown). However, when GAMs harboring 1 mg pDNAs were transplanted, we found that new bone area was markedly augmented in specimens of BMP4 and Runx2 groups compared with that in the same groups at 4 weeks (Fig. 4B,C). Absorption of β-TCP granules surrounded by new bone was progressing further, and augmented bone seemed to be mature. Mature bone tissues, which included osteocytes, were observed on the magnified micrographs in BMP4 and Runx2 groups (Fig. 4F,G). In contrast, small amounts of new bone formation were recognized in close proximity to TCP granules in the GFP group (Fig. 3E). Staining areas of osteocalcin were detected obviously in osteoblastic cells and the surface of new bone tissues in BMP4 and Runx2 groups (Fig. 4D,H). Control sections treated with preimmune serum exhibited no reactivity, indicating that the staining was specific (Fig. 4I).

**FIG. 5.**
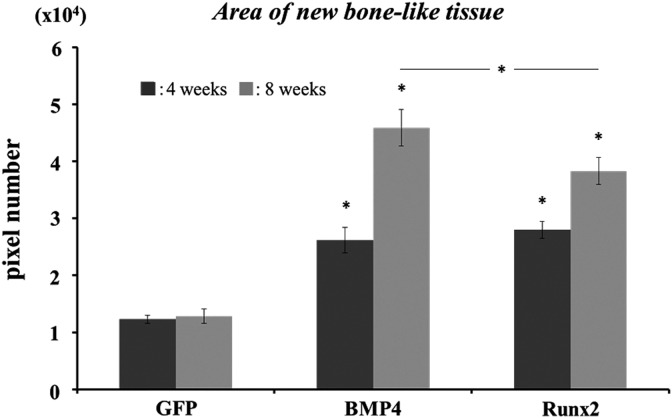
Augmented bone area at 4 and 8 weeks after GAM transplantation. Areas occupied by bone tissue on the specimens of BMP4 and Runx2 groups were increased approximately 3-fold at 4 weeks and 4–5-fold at 8 weeks compared with that of the GFP group. Asterisks represent statistical significance between GFP and other groups at each time point (*p*<0.01) and between BMP4 and Runx2 groups at 8 weeks (*p*<0.05).

Area occupied by augmented bone tissue was compared in each group in which transplanted GAM contained 1 mg of pDNAs. Areas occupied by bone tissue on the specimens of BMP4 and Runx2 groups were increased approximately 3-fold at 4 weeks and 4–5-fold at 8 weeks compared with that of the GFP group (Fig. 5). In particular, at 8 weeks, the BMP4 group showed more increased area of bone formation compared with the Runx2 group. While GAM containing only 1 mg pGFP could not show any changes of new bone area after 4 weeks, GAMs containing 1 mg pBMP4 and pRunx2 induced the further bone growth from 4 to 8 weeks.

**FIG. 6.**
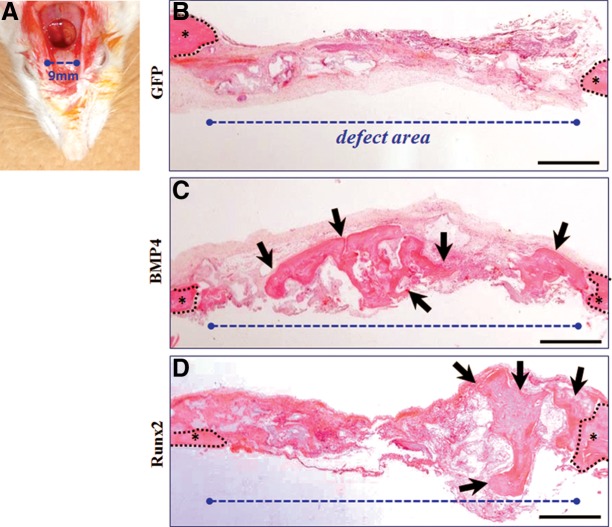
Histological observation at 8 weeks of GAM transplantation to cranial bone defect. **(A)** Gross appearance of created cranial defect with 9 mm of diameter. **(B,C)** Representative images of HE staining of specimens in GFP, BMP4, and Runx2 groups, respectively. New bone tissues were recognized ubiquitously in BMP4 **(C)** and Runx2 **(D)** groups, while bone formation was not found in the defect area of the GFP group **(B)**. Scale bar is 50 μm. Black dotted line indicates boundary of the host and defect area; asterisk, host bone; and black arrow, area of newly formed bone.

To further confirm the regenerative capability of GAMs containing 1 mg pBMP4 or pRunx2, we transplant them to the critical bone defects of rat cranium (Fig. 6A). The histology of cranial defect with 9 mm diameter at 8 weeks postoperatively is shown in Figure 6B–D. As a result, though bone formation was not detectable in the defect area of the GFP group (Fig. 6B), new bone tissues were recognized ubiquitously in that of BMP4 and Runx2 groups (Fig. 6C,D). In particular, this phenomenon seemed to be prominent in the BMP4 group.

## Discussion

This study demonstrated the usefulness of delivering GAM only containing atelocollagen and naked pDNA for bone engineering. The outcomes were as follows: (1) atelocollagen could act obviously as a carrier for delivery of genes to migrated host cells at bone surface, and (2) when loading a certain dose of pBMP4 or pRunx2, atelocollagen-based GAM could induce the prominent bone augmentation. These outcomes suggest that this simple strategy facilitates the clinical application of GAM-based bone engineering without requiring any special apparatus such as stem cells or transfection reagents/kits.

Regarding the first outcome related to matrix materials, various osteoconductive materials, including natural materials such as collagen or alginate, inorganic materials such as hydroxyapatite, and synthetic materials such as poly-glycolic acid or poly-l-lactic acid, have been attempted as matrixes of GAM to achieve bone regeneration,^14,30–33^ because these materials have been employed as carriers of growth factor delivery and/or stem cell transplantation for bone engineering.^34–36^ However, for the nonviral strategy of GAM, the matrix properties of them have been considered to show a low efficacy of transfection and require a high dose of plasmid DNA.^10,16^ For this reason, recent studies have frequently accompanied these matrixes with transfection reagents/kits, such as CaP precipitate or PEI, for nonviral GAM.^18,32^ Indeed, modified GAM using CaP precipitate, which is a kind of *in vitro* gene transfer reagent, has been reported to be able to enhance the efficiency of pDNA transfer in segmental tibial defects in rat.^20^ Moreover, several studies have recently shown the usefulness of nonviral GAM combined with PEI, which form cationic complexes with plasmids, to induce high gene expression *in vivo*.^21^

Particularly, the PEI/pDNA complex was also superior in bone regeneration on rat cranial defects. However, these reagents cause some problems regarding the safety such as the nonspecific gene expression, high cytotoxicity, or aggregation with blood components, which arose from their cationic surface charge. Therefore, a few advanced methods are examined to decrease these problems at present.^37,38^ On the other hand, administration of pDNA is considered to be safe *in vivo*, particularly when employed at a low dose. However, large amounts of the naked plasmid, such as 1–16 mg pDNAs/time, have been administrated to local sites in clinical studies for treating cancer or limb ischemia, and its safety and usefulness were suggested recently at phase I/II trial in patients.^39,40^ Therefore, we believe that *in vivo* delivery of a certain dose of naked pDNA with only matrix should be taken a fresh look as a simple and low-toxic method. Then, we are focusing on natural polymer matrix for this strategy because they are considered to be superior in biocompatibility for clinical use.

In this study, we chose atelocollagen for the matrix of GAM because the atelocollagen-mediated nucleic acid delivery system has been progressed drastically on various diseases, including cancer, autoimmune, or inflammatory diseases.^25,26,41^ As a result, 2% bovine atelocollagen showed the successful gene delivery for the required level of bone augmentation *in vivo* when contained 1 mg pDNA (dose of 6 μg/μL) per GAM. Actually, Bonadio et al. already reported that the original GAM system using the collagen matrix required more than 1 mg pDNA for inducing bone regeneration in rat. Our data may be consistent with this previous work. However, levels of new bone formation in both augmentation and defect models seem to be prominent in this study compared with the previous studies. Furthermore, a group that developed the atelocollagen-mediated gene therapy has provided 5 μg/μL, which is an optimal concentration of nucleic acid in atelocollagen for local administration.^42^ Therefore, we consider that atelocollagen can deliver this concentration of pDNA safely *in vivo* and induce the bone reliably when loaded with effective osteogenic genes. In addition, lyophilized 3D atelocollagen including β-TCP might provide the appropriate space in local sites during the time of gradual release of genes. Such plasticity of atelocollagen ought to be a very important property for GAM-based bone engineering.^43^

With regard to the effective osteogenic genes, we employed pDNAs encoding BMP4 and Runx2. The usefulness of pDNAs encoding BMP2/4 has been demonstrated in a number of studies of GAM-based bone engineering, including viral and nonviral vectors.^44^ Consistent with previous studies, atelocollagen containing pBMP4 could induce the bone formation markedly in our study. It is known that atelocollagen can release pDNA slowly for long-term with an appropriate dose in the natural body. Therefore, if our dose of pDNA in GAM is within an appropriate concentration physiologically as mentioned above, this strategy may be much safer compared with direct implantation of rBMPs. Meanwhile, although GAM containing pBMP4 could induce bone formation significantly at 8 weeks of transplantation, GAM containing pRunx2 could show the obvious bone augmentation comparably.

A previous study demonstrated that Runx2-transferred fibroblasts could not produce radiopaque regions in cranial bone defects, while BMP2-transferred fibroblasts could induce new bone formation.^45^ Therefore, this unexpected result may depend on the local circumstance of transplantation sides. In case of onlay placement, almost new bone was formed from the surface of host cranial bone. pRunx2 may be able to induce the bone formation effectively when abundant MSCs or osteogenic progenitor cells are favorable to invade GAM because MC3T3-E1 cells transfected with pRunx2 showed an increased ALP activity on the same level with pBMP4 in this study. In fact, GAM containing pRunx2 could form the bone more obviously in an augmentation model compared with a defect model. Moreover, obvious ectopic bone formation could not recognize when GAMs containing pRunx2 were transplanted to cranial defects.

In conclusion, we confirmed that atelocollagen-based GAM reliably can induce the engineered bone even for the vertical augmentation when contained a certain amount of pDNA encoding effective osteogenic proteins. Although the safety of this strategy remains unclear for clinical application, this study might support facilitating the clinical setting of GAM for bone engineering. More recently, the usefulness of the collagen–nanohydroxyapatite scaffold has been reported for bone regeneration when contained both pVEGF and pBMP2.^46^ To facilitate the clinical setting of GAM-based bone engineering, such simple strategy of nonviral GAM must be developed by clarifying the appropriate matrix and reverifying the safe concentration of pDNAs.

## Abbreviations Used

ALP: alkaline phosphatase
BMPs: bone morphogenetic proteins
CaP: calcium phosphate
GAM: gene-activated matrix
HE: hematoxylin and eosin
MSCs: mesenchymal stem cells
PEI: polyethyleneimine
TCP: tricalcium phosphate
